# Microglial Inflammatory-Metabolic Pathways and Their Potential Therapeutic Implication in Major Depressive Disorder

**DOI:** 10.3389/fpsyt.2022.871997

**Published:** 2022-06-16

**Authors:** Reza Rahimian, Claudia Belliveau, Rebecca Chen, Naguib Mechawar

**Affiliations:** ^1^Douglas Mental Health University Institute, McGill Group for Suicide Studies, Verdun, QC, Canada; ^2^Integrated Program in Neuroscience, McGill University, Montreal, QC, Canada; ^3^Department of Psychiatry, McGill University, Montreal, QC, Canada

**Keywords:** microglia, neuroinflammation, metabolic pathway, major depressive disorder, anti-inflammatory pathway, pro-inflammatory pathway, microglial pathways as therapeutic targets

## Abstract

Increasing evidence supports the notion that neuroinflammation plays a critical role in the etiology of major depressive disorder (MDD), at least in a subset of patients. By virtue of their capacity to transform into reactive states in response to inflammatory insults, microglia, the brain’s resident immune cells, play a pivotal role in the induction of neuroinflammation. Experimental studies have demonstrated the ability of microglia to recognize pathogens or damaged cells, leading to the activation of a cytotoxic response that exacerbates damage to brain cells. However, microglia display a wide range of responses to injury and may also promote resolution stages of inflammation and tissue regeneration. MDD has been associated with chronic priming of microglia. Recent studies suggest that altered microglial morphology and function, caused either by intense inflammatory activation or by senescence, may contribute to depression and associated impairments in neuroplasticity. In this context, modifying microglia phenotype by tuning inflammatory pathways might have important translational relevance to harness neuroinflammation in MDD. Interestingly, it was recently shown that different microglial phenotypes are associated with distinct metabolic pathways and analysis of the underlying molecular mechanisms points to an instrumental role for energy metabolism in shaping microglial functions. Here, we review various canonical pro-inflammatory, anti-inflammatory and metabolic pathways in microglia that may provide new therapeutic opportunities to control neuroinflammation in brain disorders, with a strong focus on MDD.

## Introduction

An association between inflammation and major depressive disorder (MDD) has long been hypothesized based on investigations using various approaches. Studies have reported elevated levels of both peripheral (1) and central (2–7) pro-inflammatory cytokines in depressed patients, supporting the hypothesis of an immune-mediated etiology of MDD (8–10). Indeed, subsets of MDD patients have increased concentrations of circulating cytokines such as tumor necrosis factor-alpha (TNF-α) and interleukin (IL)-6 (11) and increased expression of innate immune-related genes in the blood. Also, bidirectional relationships between depression and inflammatory or autoimmune disorders exist. Namely, there is a high incidence of co-morbid inflammatory disease and rheumatoid arthritis (RA) in MDD patients (12).

One of the most important cell types involved in regulating neuroinflammation are microglia, which can modulate immunological responses and play a fundamental role in maintaining homeostatic brain functions. The implication of microglia in normal brain physiology includes, but is not limited to, synaptic pruning, phagocytosis, oligodendrocyte maturation and neurogenesis (10, 13). Functionally, microglia are one of the most diverse cell types in the central nervous system (CNS), as they dynamically adapt, at both the cellular and molecular levels, to their ever-changing environment (10, 14). The heterogeneous nature of microglia has been highlighted by high-throughput approaches like single cell RNA-sequencing. Indeed, factors such as brain region, sex, age and type of pathology can significantly affect microglial phenotype, including gene expression signatures and secretory profiles (15, 16). Under physiological and pathological conditions, microglia display spatial heterogeneity in density, morphology, turnover rate, pruning, metabolism and molecular signature (17–19).

Microglial activation occurs through inflammatory insult or slight alterations in brain homeostasis. This activation is dependent on the context and the type of stressor or pathology. Microglia determine the pathological outcome of stressors through secretion of cytokines, chemokines and growth factors and psychopathologies have repeatedly been associated with long-lasting priming and sensitization of cerebral microglia (10, 20, 21). Microglia also modulate communication between the nervous and the immune system in response to different physiological, psychological and immunological stressors. They are in fact considered to be responsible for the decreased neuroplasticity observed in depression (22) and recent findings have associated microglial abnormalities with neuropsychiatric disorders such as MDD, which have been termed microgliopathies by some (23–25).

Manipulating the microglial phenotype is an intriguing strategy for developing new therapeutics for MDD. Particular attention is currently given to exploiting alternative microglial polarization as a potential therapeutic option in a wide range of neurological and neuropsychiatric disorders (15). To achieve this, canonical pathways that govern tuning of the microglial phenotype have been investigated including transforming growth factor β (TGF-β), IL-4 receptor and peroxisome proliferator-activated receptors-gamma (PPAR-γ) (26, 27). We and others previously published reviews on the roles played by microglia in psychiatric disorders (10, 13, 21, 28). However, a review focused on different canonical microglial pro-inflammatory, anti-inflammatory and metabolic pathways and their translational value in drug discovery for MDD is lacking. We aim to fill the gap.

## Role of Neuroinflammation in Psychopathology

The role(s) played by the innate immune response in MDD has been the object of several experimental and clinical studies in which microglia have increasingly become the focus of investigation. Microglia have been studied in different psychiatric disorders using various approaches, including postmortem investigations in humans as well as experimental studies in animal models (21, 29, 30). The term neuroinflammation, denoting inflammatory processes in the CNS, is a rather general notion that could include both peripheral and central components of inflammation. Interestingly, a number of studies have suggested the involvement of peripheral inflammation in the pathogenesis of depression. Menard et al. associated a reduced expression of the endothelial cell tight junction protein claudin-5 with abnormal blood vessel morphology in the nucleus accumbens of stress-susceptible but not resilient mice (31). In a more recent study, the same group showed that epigenetic regulation of claudin-5 is associated with stress resilience. Indeed, they identified nuclear factor kappa-light-chain-enhancer of activated B cells (NFκB) signaling pathway and histone deacetylase 1 as mediators of stress susceptibility. Pharmacological inhibition of histone deacetylase 1 rescued claudin-5 expression in the nucleus accumbens and promoted resilience (32). It is to be noted, however, that blood–brain barrier (BBB) disruption is not a common phenomenon in animal models of depression (31) and that the mechanism underlying this region-specific BBB abnormality remains to be clarified.

Recently, neuroinflammation has been used to explain functional microglial abnormalities observed in psychopathologies. A developed mouse model of obsessive-compulsive disorder serves as an interesting example of the link between microglial abnormalities and mental illness. Mutant *HOXB8* mice display unexpected behavior manifested by compulsive grooming and hair removal (33). These actions directly mirror trichotillomania seen in humans with obsessive-compulsive spectrum disorder (33). Chen et al. reported that, in the brain, the *HOXB8* cell lineage exclusively labels bone marrow-derived microglia. This finding strongly fosters the theory that the excessive grooming behavior observed in *HOXB8* mutant mice is a consequence of defective microglia, thus relating hematopoietic function to mouse behavior (33). Another interesting example is the effect of CX3CR1 deficiency on behavior relevant to post-traumatic stress disorder. Neuronal CX3CL1 and its microglial target CX3CR1 play an essential role in synaptic plasticity and a correlation between CX3CR1 deficiency and increased fear behavior as well as an anxiolytic-like phenotype have been reported by Schubert et al. (34). However, Milior et al. observed a contradicting finding in which microglial CX3CR1 knock-out mice were resilient to chronic unpredictable stress (CUS) suggesting microglia-neuron communication may be at the interplay of resilience or susceptibility to a depression-like phenotype (35). Colony-stimulating factor 1 receptor signaling was also shown to control cerebellar microglia and to be essential for motor function and social interaction (36).

## Importance of Microglia in Major Depressive Disorder

Despite a strong correlation between microglial activation and depression in pre-clinical and clinical studies, it remains unclear whether microglial abnormities play a causal role in depression (37). Clinical findings indicate a strong correlation between disease etiology and inflammation (37). Subsets of MDD patients consistently display increased levels of pro-inflammatory cytokines such as TNF-α and IL-6 (11, 30). A previous postmortem investigation by our group has shown that the percentage of primed microglia is increased in the dorsal anterior cingulate cortex (ACC) of depressed suicides compared to matched controls (38). This observation is consistent with independent reports of microglial activation in the ACC of MDD patients (39).

There is a growing body of literature showing increased microglial activation in inflammatory and non-inflammatory rodent models of depression. The most studied inflammatory model is that of lipopolysaccharide (LPS) injection. Systematic LPS administration not only triggers peripheral immune responses but also activates microglia in the brain (37, 40). Following LPS challenge, pro-inflammatory cytokines such as TNF-α, IL-1β, and IL-6 are upregulated in different brain areas (41, 42). These inflammatory changes are accompanied by decreased sucrose preference and increased immobility in the forced swim test (43). Moreover, animals that receive acute or chronic non-inflammatory stress also show microglial activation along with morphological changes and increased levels of pro-inflammatory cytokine release in different brain regions including the hippocampus, thalamus and prefrontal cortex. For example, mice subjected to chronic social defeat (CSD) exhibit increased numbers of CD68-expressing microglia that have increased phagocytic capacity. Several other groups have reported microglial dysregulation following CSD stress (44–49). Lehmann et al. have reported that depressive-like behavior throughout and following CSD involves microglia-derived reactive oxygen species (ROS). Using colony stimulating factor receptor antagonist PLX5622 to deplete microglia before and during the 14-day CSD procedure, mice were protected from the effects of stress as measured by light/dark and social interaction paradigms (10, 50). More evidence indicating the involvement of microglial activation in depression comes from minocycline studies. Minocycline is an anti-inflammatory tetracycline that inhibits microglial activation and subsequent neuroinflammation (37, 51). Minocycline treatment does not have anti-depressive behavioral effects in naïve mice (52), however, it elicits significant anti-depressive effects in the rat model of chronic unpredictable mild stress (CUMS) (53). Intriguingly, combinatorial therapy of minocycline with antidepressants provides better clinical outcomes in some MDD patients, implying the contribution of neuroinflammation and microglial activation in a subset of patients afflicted with this psychopathology (54).

In the following sections, we aim to highlight the potential roles played by microglia in the pathogenesis of MDD, with a focus on the roles played by the canonical pro-inflammatory, anti-inflammatory and metabolic pathways.

## Pro-Inflammatory Pathways in Microglia

### Tumor Necrosis Factor-α Mediated Pathway

TNF-α is a trimeric cytokine that is expressed either in a 27 kDa transmembrane form or a 17 kDa soluble form processed by TNF-α converting enzyme (55–57). TNF-α exerts its pleiotropic effects by binding to two primary receptors: TNF-α Receptor 1 (TNFR1) and TNF-α Receptor 2 (TNFR2). Soluble TNF-α typically binds TNFR1 after clustering at the cell membrane (58, 59). Transmembrane TNF-α preferentially binds TNFR2 as a ligand and can serve as a receptor for cell-to-cell contact (56, 59). Regardless of form, after a TNF-α ligand binds, TNFR1 and TNFR2 from homodimers to induce downstream signaling for cellular processes such as defense from foreign pathogens, enhancing inflammation and promoting cell survival or apoptosis (57, 60, 61).

TNF-α is produced by different cell types in the CNS, including neurons, astrocytes, microglia and endothelial cells (62, 63). However, monocytic immune cells like microglia are the dominant secretors and targets of TNF-α (57). TNF-α is now well understood as a critical pro-inflammatory cytokine that has an instrumental roles in the CNS, including innate immunity, sleep regulation, neuronal activity and necrotic and apoptotic cell death (58, 64). Under physiological conditions TNF expression is induced by basal activity in microglia, neurons and astrocytes. This cytokine is essential for regulating neuronal function including synaptic activity. Neurons constitutively express TNF-α receptors, which are important for mediating neuroprotection against neurotoxic stimuli (65).

Microglia-derived TNF-α is critical in innate immune responses within the CNS (66). Previous studies have documented the ability of TNF-α to influence microglial function in response to neuroinflammatory insults. For instance, to achieve a swifter recognition of foreign pathogens, TNF-α binds to its microglial receptors and upregulates the expression of toll-like receptor (TLR) 2, a pattern recognition receptor specialized for bacteria, enhancing microglia’s overall immune response (67). Aside from priming microglia for enhanced pathogen detection, this cytokine also enhances natural killer cells’ and macrophages’ ability to kill cells and phagocytose, respectively (58, 67).

TNFR1 and TNFR2 notably have varying functions within the brain (Table 1), some of which are region-specific. It was previously observed that the reparative function of TNF-α on neurons in the striatum is reliant upon TNFR1 (68). At the same time, similar capabilities of TNF-α in the hippocampus depend upon TNFR2 but the receptors’ expressions were equal in both regions (68). A more recent study found similar effects when investigating TNFR1 and TNFR2 single nucleotide polymorphisms (69). These results indicated that TNF-α regulation of striatal morphology was predominated by TNFR1 signaling while regulation of hippocampal morphology was shown to rely primarily on TNFR2 (69). It is thought that this region-specificity may be due to the two receptors’ differential impacts on cell survival since TNFR1’s downstream processes promote apoptosis and TNFR2’s pathways are more anti-apoptotic (68).

**TABLE 1:** Overview of studies on TNFR1 and TNFR2’s differential processes within the brain.

Study	Study performed	TNFR type	Cell type	Observed effects
(68)	TNFR1 and TNFR2 levels measured in encephalitis mouse model	TNFR1	Striatal Neurons	TNFR1 carries out reparative function of TNF-α on striatum neurons
		TNFR2	Hippocampal neurons	TNFR2 carries out reparative function of TNF-α in hippocampus
(69)	Single nucleotide polymorphisms and associated brain morphology changes	TNFR1	Striatal neurons	TNFR1 responsible for TNF-α regulation of striatal morphology through apoptosis
		TNFR2	Hippocampal neurons	TNFR2 responsible for TNF-α regulation of hippocampal morphology through anti-apoptotic processes
(87)	Anti-TNF-α therapy and its effects on cell survival	TNFR1	Cholinergic neurons	When TNFR1 receptor blocked by anti-TNF-α, reduced apoptosis
		TNFR2	Cholinergic neurons	When TNFR2 blocked by anti-TNF-α, reduced cell protective processes
(88)	TNFR2 KO mice	TNFR2	Microglia	KO mice displayed early onset experimental autoimmune encephalitis
(89)	Activating TNFR2 in cultured mouse microglia	TNFR2	Microglia	TNFR2 regulates production of pro-regenerative and neuroprotective factors such as granulocyte colony-stimulating factor and IL-10

*TNF-α, tumor necrosis factor-alpha; TNFR1, TNF-α receptor 1; TNFR2, TNF-α receptor 2; KO, knockout; IL-10, interleukin-10.*

**FIGURE 1:**
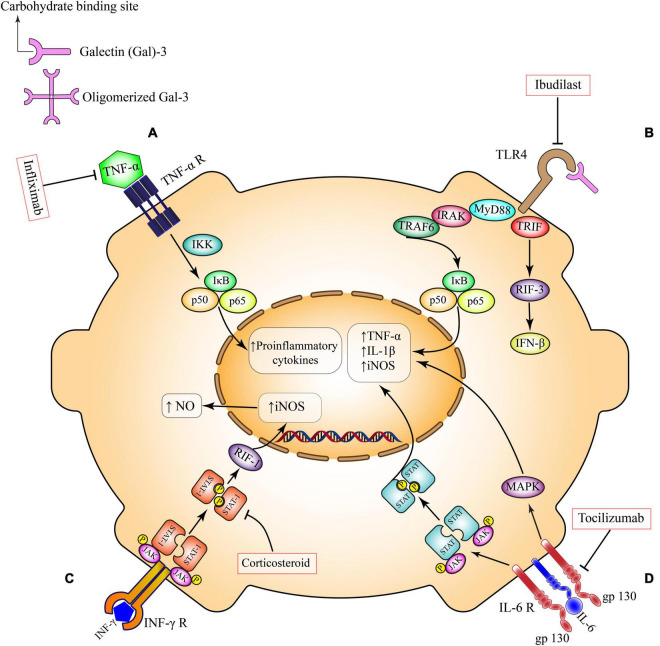
Pro-inflammatory pathways in microglia. **(A)** TNF-α receptor activation, induces the canonical pro-inflammatory transcriptional factors such as NFκB and subsequent production of inflammatory mediators. This pathway can be inhibited by Infliximab. **(B)** TLR4 ligands and secreted Gal-3 directly bind to TLR4 on the microglial surface and exacerbates inflammatory responses through induction of different cytokines and chemokines. This pathway can be inhibited by Ibudilast. **(C)** Activation of INF-γ receptor on microglia triggers the overexpression of inducible nitric oxide synthase (iNOS) and overproduction of nitric oxide via Janus kinase (JAK)/signal transducer and activator of transcription (STAT)/RIF-1 pathway. Corticosteroids can suppress this pathway at the level of STAT factors. **(D)** IL-6 *trans*-signaling occurs in brain cell types that have membrane bound gp130, including microglia. IL-6 bound to soluble IL-6R activates signaling through membrane bound gp130. This *trans*-signaling is thought to be pro-inflammatory via the induction of JAK/STAT and MAPK signaling pathways. Tocilizumab inhibits this pro-inflammatory pathway. TNF-α, tumor necrosis factor-α; IKK, the IκB kinase; NO, nitric oxide; iNOS, inducible nitric oxide synthase; IL-1β, interleukin 1 beta; IL-6, interleukin 6; JAK, Janus kinase; STAT, signal transducer and activator of transcription; RIF-1, replication timing regulatory factor 1; MAPK, a mitogen-activated protein kinase; TLR4, toll-like receptor 4; MYD88, myeloid differentiation primary response 88; TRIF, TIR-domain-containing adapter-inducing interferon-β; IRAK, interleukin 1 receptor associated kinase; TRAF, TNF receptor associated factor; IFN-β, interferon-β; IFN-γ, interferon gamma.

The distinct effects of these receptors on cell survival and inflammation can be understood through their different recruitment of signaling complexes after a ligand binds (Figure 1). Active TNFR1 homodimers allow four different complexes to form to engage various cellular processes (70). Complexes I, IIa, and IIb similarly activate NF-κB and mitogen-activated protein kinases (MAPK) to promote cell survival, cell proliferation, immune defense and inflammation (70). Complexes IIa and IIb are additionally responsible for activating the caspase apoptotic pathway (70). The last complex, complex IIc, also plays a role in inflammation but is most notable for its role in necroptosis (70). Unlike TNFR1 pathways, the current understanding of TNFR2 is not as comprehensive (71). However, previous studies with TNFR2 knockout mice have shown its importance in anti-inflammatory and cell-protective processes (71). Furthermore, it is theorized that active TNFR2 homodimers recruit adapter protein TNF receptor-associated factor 2 to activate NF-κB pathways (71). Despite TNFRs sharing similar structures and ligands there is a clear heterogeneity in their downstream effects.

TNF-α has long been implicated in several peripheral and central inflammatory conditions (58, 72, 73). Meanwhile, research on the role of TNF-α in psychiatric disorders is evolving. In this context, TNF-α’s pro-inflammatory functions may exacerbate or contribute to depressive symptoms (74). Both TNFR1 and TNFR2 pathways can modulate inflammatory pathways through downstream NFkB signaling (60). Additionally, TNF-α can induce glutamate-mediated excitotoxicity. This cytokine facilitates crosstalk between microglia and astrocytes to promote the release of astrocytic glutamate, the formation of excitatory synapses and the release of more TNF-α from microglia (75). Therefore, TNF-α mediated pathways can lead to extraneous inflammation and cell death contributing to the worsening of MDD.

In accordance, several studies have seen higher levels of serum TNF-α associated with depressive symptoms and in MDD patients compared to matched controls (76–78). A few studies measuring cytokine profiles in cerebrospinal fluid (CSF) have not found differences in TNF-α levels between depressed patients and healthy controls (79). However, there are only few such studies of CSF cytokine levels and the results seem rather inconsistent as compared to studies of peripheral (plasma) cytokines (79). In addition, plasma cytokine profiles do not necessarily represent cytokine levels within the CNS (80).

In a pioneer study, Ohgidani et al. showed that acute stress induces TNF-α secretion from hippocampal microglia resulting in mouse working memory deficits. These authors observed that morphological changes in hippocampal microglia did not occur (81). Furthermore, etanercept, a TNF-α inhibitor, rescued the working memory impairment accompanied by a reduction in hippocampal TNF-α (81). Indeed, maladaptive microglial activation may be linked to MDD and modulating microglial activation seems a promising therapeutic target for depression.

A previous study with monoclonal antibody against TNF-α revealed that this intervention quelled symptoms of anhedonia but did not affect depression scores significantly compared to placebo groups (82). Current data seem to support the effectiveness of anti-inflammatory agents as antidepressants only in patients with increased peripheral inflammation (83). This subgroup may include MDD patients with increased inflammatory markers or those with medical conditions characterized by increased levels of peripheral inflammation (84). Specifically, anti-TNF-α therapy was found not to be effective in all treatment-resistant depression patients, but it did improve depressive symptoms in those with higher baselines of inflammatory markers (85). One major reason for such failure is the double-edged role of microglial TNF-α in fundamental physiological processes such as the neuroinflammatory response to tissue damage, neuronal circuit formation, synaptic plasticity and myelin degeneration and repair (86). Indeed, any positive impact from blocking TNFR1 receptors would be nullified by blocking TNFR2 activation, adding another complex layer upon possible therapies for MDD (87). It has been shown that the TNF-α mediated activation of microglial TNFR2 is instrumental for the protective functions of these cells (86). For instance, microglia-specific TNFR2 knockout mice display early onset of experimental autoimmune encephalitis (88). In this context, it has been shown that TNFR2 regulates the production of pro-regenerative and neuroprotective factors including granulocyte colony-stimulating factor and IL-10 in microglia (89). It is noteworthy that protective aspects of TNF-α signaling in microglia have been greatly overlooked with respect to the pro-inflammatory ones. Thus, more investigations are needed to decipher the mechanisms regulating the balance between TNFR1 and TNFR2 pathways in microglia in order to limit the detrimental immune responses without blocking the protective ones (86).

### IL-6 Mediated Pathway

Over the years, the cytokine IL-6 has been linked to stress-related disorders such as depression and anxiety (90). This cytokine is a small multifunctional protein (91) that can be produced by several cell types including endothelial cells, epithelial cells, astrocytes, microglia and neurons (92, 93). IL-6 belongs to a family of proteins that utilize glycoprotein 130 (gp130) as a signal transducer (Figure 1). Depending on the presence of IL-6 receptor (IL-6R) or membrane bound gp130 which are expressed differently in different cell types, IL-6 has pro- or anti-inflammatory properties (93) resulting in either inflammatory or anti-inflammatory cascades (90).

According to a few major meta-analyses, IL-6 is one of the most consistently elevated cytokines in the blood of patients with MDD (11, 94, 95). Remarkably, IL-6 blood levels might have a predictive value as a biomarker. Moreover, peripheral levels of IL-6 correlate with symptom severity of antidepressant non-responders (96). In addition, increased levels of IL-6 have been reported in the CSF of patients with MDD as well as in suicide attempters (3, 97). Unfortunately, research addressing the role of microglial derived IL-6 are lacking in human postmortem studies. Only one pioneer study indicates a non-inflammatory phenotype of microglia in MDD following single-cell mass cytometry of microglia (98). The authors performed single-cell analysis of microglia from four different postmortem brain regions including frontal lobe, temporal lobe, thalamus and subventricular zone of medicated individuals with MDD and they found no evidence for the induction of canonical pro-inflammatory (IL-1β, IL-6, and TNF-α) and anti-inflammatory cytokines such as IL-10 (98).

Various pre-clinical studies have investigated the role of microglial IL-6 in the context of stress. For instance, increased IL-6 mRNA is found in microglia isolated directly from the brains of mice that have undergone repeated social defeat (RSD) stress (99) and treatment with the antidepressant imipramine inhibits social avoidance behavior and diminished microglia IL-6 in mice exposed to stress (90, 99). In another study, Aniszewska et al. found that stress induced a significant increase in the number of IL-6-immunoreactive microglia in the hippocampus, cortex and brain stem (100).

Blocking IL-6R-mediated pathways (e.g., tocilizumab) or neutralizing IL-6 function (e.g., sirukumab) might have clinical value in a subset of MDD patients, especially in treatment-resistant cases or in patients with peripheral inflammatory diseases (101). Interestingly, the efficacy of interleukin-6 neutralizing antibodies on symptoms of MDD patients with RA has been reported (102). However, BBB penetration and adverse effects may limit their use in MDD patients without the history of peripheral inflammatory diseases such as RA (103). In addition, there are two types of IL-6 signaling: a classical anti-inflammatory signaling and a *trans*-signaling proinflammatory signaling. It means that general targeting of IL-6 pathways in MDD either with IL-6R inhibitors or IL-6 blocker is not an optimal choice (101, 103). A more selective intervention seems more promising for future drug discovery targeting IL-6 signaling (101).

### Toll-Like Receptor 4 and Nuclear Factor-Kappa B Mediated Pathways

TLR4 is one of nine members in the TLR family of pathogen-specific pattern recognition receptors dedicated to responding to unique structural components of foreign microbial agents to trigger immune responses (104, 105). As such, this receptor is highly involved in regulating brain innate immune responses in pathophysiological conditions. TLR4 is notably the most researched receptor within the TLR family. It is well known to be primarily responsible in the reaction against Gram-negative bacteria by binding to LPS, its pathogen-associated molecular pattern (106). However, TLR4 has also been shown to recognize damage-associated molecular patterns (DAMPs) and xenobiotics (107). Additionally, TLR4 can be activated by other non-bacterial TLR4 agonists naturally present within the body such as saturated fatty acids (108). TLR4s are generally expressed in myeloid lineage cells like macrophages and other non-immune cells like endothelial cells (106). Within the CNS, TLR4 is expressed primarily by microglia but can also be found in astrocytes, oligodendrocytes and neurons (107).

NF-κB refers to a group of transcription factors that serve as significant regulators of pro-inflammatory genes and has been of particular interest as a target for pharmacological treatments in inflammatory diseases (109). They exist as two subfamilies of inducible dimers, made up of either DNA binding proteins from the ‘Nκ-kB’ or ‘Rel’ family, which can then form homodimers or heterodimers (110). Alternatively, the term NF-κB can also describe p50-RelA heterodimer, the predominant NF-κB dimer present in many cells (111). Regardless, all NF-κB dimers are constitutively inhibited by IkB proteins that are degraded to activate and translocate the transcription factor into the nucleus (109). NF-κB activity is rapidly induced in microglia following inflammatory insults (112).

During activation with LPS, the pathogen-associated molecular pattern forms a multimolecular complex with TLR4 and its accessory molecules which readies the receptor for dimerization (113). The formed dimer then induces downstream effects (Figure 1) either through the myeloid differentiation primary response 88 (MyD88)-dependent pathway or the MyD88-independent pathway (106, 114). The MyD88-dependent pathway leads to activation of transcription factors that induce the expression of pro-inflammatory cytokine genes (114). The MyD88-independent pathway, also known as the TIR-domain-containing adapter-inducing interferon-β dependent pathway, also activates transcription factors but mediates the induction of type 1 interferon-inducible genes (114). In both cases NF-κB is activated (115).

TLR4 and NF-κB are of particular interest in pathophysiological conditions due to their significant roles in innate immunity. Accordingly, many of the following studies have implicated their dysregulation within various neurodegenerative diseases and psychiatric disorders (116–118). Elevated TLR4 expression and associated overactive microglia were observed within a transgenic mouse model of Alzheimer’s Disease (AD) leading to cognitive impairment (119, 120). Furthermore, previous investigations have also characterized an increase in NF-κB activation within the CNS of animal models and patients with neurodegenerative disorders (117). Interestingly, a study of postmortem brains from patients with schizophrenia revealed that TLR4 levels are increased within the cerebellum but decreased within the prefrontal cortex (PFC) suggesting that general dysregulation, rather than upregulation, could lead to harmful effects (118). In the same study, NF-κB levels were notably altered inversely to TLR4 levels, with increased levels within the PFC and decreases in the cerebellum (118). Another investigation demonstrated increases in NF-κB levels, especially in microglia, as a neuroinflammatory mechanism in autism spectrum conditions (116). The studies above support the idea that TLR4, NF-κB and their combined pathway likely play significant roles in neuroinflammatory response in MDD. More specifically, the dysregulation of TLR4 and NF-κB inflammatory processes has been suggested to be involved in MDD. TLR4 single gene polymorphisms were associated with suicide and anxiety scores in MDD patients, while methylation levels of TLR4-associated CpGs were related to the severity of depressive symptoms (121, 122). In general, increased TLR4 expression and decreased expression of its inhibitor TNFAIP3 were associated with depressive symptoms (107). Similarly, increased NF-κB activity also appears to play a role in depression. In a rat early-life stress model, the depression-susceptible animals generally displayed more activated NF-κB. Inversely, the subgroup showing resilient phenotype had more inactivated NF-κB (123). Another notable study supported this idea by demonstrating improved depressive-like behaviors in mice when increased NF-κB expression was inhibited (124). Other studies have specifically investigated TLR4-NF-κB pathways in depression. A supporting study indicated that suppressing the TLR4-NFκB signaling pathway inhibits depression-like behavior in mice (125). These studies sparked further interest in investigating these pathways to develop new antidepressant candidates for MDD patients (Table 2).

**TABLE 2:** Overview of studies performed to investigate TLR4 and NF-κB’s roles in neuropsychiatric disorders.

Study	Method	Condition of Brain/Behavior	Finding
(118)	Measure TLR4 and NF-κB levels in human postmortem brains	Schizophrenia	PFC: (1) ↓ TLR4, (2) ↑ NF-κB Cerebellum: (1) ↑ TLR4, (2) ↓ NF-κB
(116)	Measure NF-κB levels in postmortem human brains	Autism spectrum conditions	↑ NF-κB levels especially in microglia
(121)	Associative study on TLR4 single gene polymorphisms and MDD	Individuals with higher suicide and anxiety scores	↑ TLR4 single gene polymorphisms
(122)	Associative Study of epigenetic effects of TLR4 on severity of symptoms	Higher severity of depressive symptoms	↑ methylation levels of TLR4-associated CpGs
(123)	Measure levels of active NF-κB in an early-life stress rat model	Depression susceptible phenotype	↑ activated NF-κB
		Depression resilient phenotype	↑ inactivated NF-κB
(124)	NF-κB inhibited in mice model to see behavioral changes	Improved depressive-like behaviors	↓ overactivated NF-κB
(125)	Suppressing TLR4-NF-κB pathway in mice	Inhibits depression-like behavior	↓ signaling of TLR4-NF-κB pathway

*↑, increased; ↓, decreased; TLR4, toll-like receptor 4; NFκB, nuclear factor kappa-light-chain-enhancer of activated B cells; PFC, prefrontal cortex.*

The current literature strongly supports the existence and importance of TLR4, NF-κB and their conjoined roles in MDD. Although TLR4 activation seems to play a role in MDD, the underlying mechanisms are unclear. Much of the previous work implicating TLR4 in MDD have primarily focused on the role of TLR4 following activation by LPS in bacterial infections (114). However, as noted previously, TLR4 can be triggered by many different ligands; thus, current perspectives are limiting and require further investigation from novel angles (106–108). More specifically, it may be useful to consider the immune signaling that coincides with other events aside from infection, such as during the critical period of development or epigenetic modifications (126, 127). These are important time points that may impart predisposing factors on individuals if TLR4 signaling is altered (126, 127). Furthermore, these deleterious inflammatory states associated with altered TLR4 pathways may also be attributed to other mechanisms such as neuroendocrine signaling and dysregulated gut microbiota (127). Therefore, to improve the efficacy of future drugs targeting TLR4 in MDD, research into more diverse mechanistic facets should be conducted. A recent study used this approach to propose polyphenols as a potential group of drug candidates for future consideration (128).

### Interferon-Gamma Mediated Pathway

Interferon-gamma (IFN-γ) is a pleiotropic soluble cytokine that is produced by different immune cell types including lymphocytes, B cells and antigen-presenting cells. In the CNS, different cells such as neurons, microglia and astrocytes produce this cytokine and express its receptors. The activation of the IFN-γ receptor induces several canonical downstream pathways (Figure 1) such as the janus kinase (JAK) 1 and 2, signal transducer and activator of transcription (STAT) 1 and the extracellular-signal-regulated-kinase (ERK) 1/2. Many genes, as well as micro RNAs and long non-coding RNAs, are activated following IFN-γ receptor stimulation (129). Neuroinflammation mediated by IFN-γ has been reported in neurological disorders. However, the effects of IFN-γ on behavior in the context of stress are mostly unknown (129). Intriguingly, IFN-γ knockout mice show decreased anxiety- and depressive-like behaviors (129). These effects are accompanied by elevation of serotonergic and noradrenergic activity in the central amygdaloid nucleus, together with increased baseline plasma corticosterone, decreased neurogenesis in the hippocampus and decreased levels of nerve growth factors in the PFC, indicating that IFN-γ modulates anxiety and depressive states and is involved in CNS plasticity (130–132). In an intriguing report, Zhang et al. showed that intracerebroventricular injection of IFN-γ in mice causes impairment of adult hippocampal neurogenesis, behavioral despair, anhedonia and cognitive loss. Furthermore, IFN-γ induces microglial activation that is associated with morphological changes and upregulation of phagocytic marker CD68 and pro-inflammatory cytokines (IL-1β, TNF-α, and IL-6) (133). Inhibition of the JAK/STAT1 pathway, downstream of IFN-γ receptor, suppresses microglial-mediated neuroinflammation, diminishes depressive-like behaviors and improves memory (133).

An array of studies has demonstrated the possible effects of antidepressants, particularly selective serotonin reuptake inhibitors (SSRIs), on inflammatory responses and microglial function (10, 134). Besides their known therapeutic mechanism involving the modulation of the serotonergic system, SSRIs can regulate the activation state and secretory profile of microglia (10). In this context, Horikawa et al. reported that paroxetine and sertraline prevent microglial activation by inhibiting IFN-γ-induced elevation of intracellular calcium (135). Interestingly, Alboni et al. found that quality of the environment effects the mechanism of action of Fluoxetine. Enriched environments coincident with Fluoxetine administration induced pro-inflammatory microglial profiles while a stressful environment resulted in anti-inflammatory secretory profiles (134).

It is established that microglia adopt reactive states in response to an inflammatory insult. However, at both transcriptional and functional levels, microglia appear to be more complex and dynamic than anticipated. This might explain why engagement of microglia can be either neuroprotective or neurotoxic, leading to attenuation or exacerbation of disease progression (10, 15, 136) depending on the context. According to the traditional classification of macrophages/microglia, during microglial activation following an inflammatory insult, cell morphology is altered either to M1, the typically activated phenotype, or to M2, an alternative activated phenotype; and this phenotypic switch depends on the type of insult. M1 microglia are considered proinflammatory and produce mediators such as TNF-α and IL-1β. It has been shown that INF is a canonical cytokine that can polarize microglia toward M1. It is noteworthy that the classification of the M1 and M2 phenotypes have been challenged (10, 136, 137). The reason is that such classification has been defined mainly based on *in vitro* studies of peripheral macrophages and that M1 and M2 states fail to emerge in brain resident microglia. It is now accepted that activated microglia co-express canonical gene products associated with both M1 and M2 states. Indeed, following brain injury, microglia do not simply switch to a polarized “M1-only” or “M2-only” phenotype but rather display a mixed phenotype due to the complex signaling cascades surrounding them (27, 136, 137). However, in the context of this review, we will continue to use the broad categories of activated pro-inflammatory and anti-inflammatory unless the study mentioned investigated more dynamic phenotypes.

The choroid plexus (ChP), a highly vascularized tissue that produces CSF and lacks a BBB, is an interface between peripheral and central immune responses (138). Our group previously investigated the cellular and molecular inflammatory profile of the ChP of the lateral ventricle in depressed suicides and healthy controls (138). We measured the content of several pro- and anti-inflammatory transcripts as well as the density of Iba1^+^ macrophages associated with the ChP epithelial cell layer. The levels of pro-inflammatory markers, ICAM1 (a protein implicated in immune cell trafficking) and Iba1, were measured to be significantly downregulated in depressed suicides as compared to controls (138). Intriguingly IFN-γ signaling has been shown as a selective key regulator of immune cell trafficking across the ChP epithelium under physiological conditions of CNS immune surveillance and following neuroinflammatory insult (139). This unique mechanism could be harnessed to adjust the interplay between the peripheral immune system and microglia in affective disorders.

## Anti-Inflammatory Pathways in Microglia

### Alpha-Seven Nicotinic Receptor Mediated Pathway

The alpha-7 nicotinic acetylcholine receptor (α7 nAChR) is a ligand-gated ion channel expressed by macrophage/microglia (140, 141) and has proved to be a promising target in pharmacotherapy of psychiatric disorders. α7 nAChR agonists or partial agonists are known to improve cognitive dysfunction by regulating microglial activation through inhibition of canonical pro-inflammatory transcriptional factors such as NFkB and induction of anti-inflammatory signaling pathways such as nuclear factor-erythroid factor 2-related factor 2 (Nrf2) (142–144). In fact, there is ample evidence that the cholinergic system plays a fundamental role in regulating central inflammation and glial activation via homomeric α7 nAChRs (145–147). The α7 nAChRs consist of five α subunits and are expressed by neuronal and glial cells (148, 149). These ligand-gated ion channels allow for calcium influx and subsequent ultra-rapid desensitization (150, 151). α7 nAChRs are widely expressed in the brain, including in regions such as the PFC, hippocampus and other limbic areas (150). Microglial α7 nAChRs play important roles in regulating inflammatory processes in the CNS (148, 150). Stimulation of α7 nAChR leads to a reduction in glial activation and decreases in proinflammatory cytokine levels in different brain regions (152–154).

**FIGURE 2:**
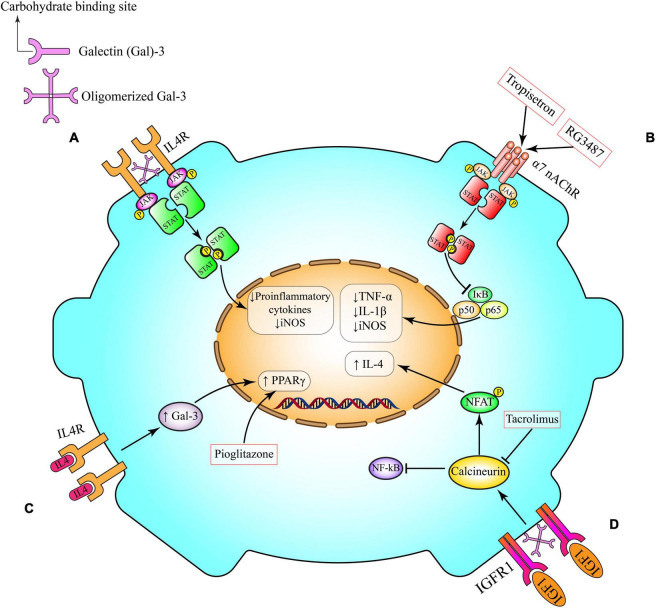
Anti-inflammatory pathways in microglia. **(A)** Gal-3 induces alternative microglia activation through interaction with IL-4 receptor (IL4R). Following Gal-3 lattice formation the carbohydrate-binding site of Gal-3 molecules interacts with glycosylated IL4R and prevents their endocytosis and also over activation of IL4R and its anti-inflammatory signaling. **(B)** Activation of α7 nAChR on microglia triggers anti-inflammatory cascades, including Janus kinase (JAK)/signal transducer and activator of transcription (STAT) and PI3K/Akt, which potentiate the activity of transcriptional factor Nrf2 and its downstream pathways (HO-1 and CAT), and inhibits the canonical proinflammatory protein NFκB, which governs the production of proinflammatory cytokines (e.g., TNF-α) and enzymes (e.g., iNOS and COX-2) involved in neuroinflammation. This pathway can be induced by tropisetron and RG3487. **(C)** IL-4 can interact with its tyrosine kinase IL4R on microglia cell surface. This interaction might activate one of the canonical transcriptional factors that are involved in microglia polarization such as PPAR-γ. Pioglitazone activates PPAR-γ. **(D)** By crosslinking insulin-like growth factor 1 receptor (IGFR-1), secreted Gal-3 will prevent early endocytosis and over-activate the Janus kinase (JAK)/signal transducer and activator of transcription STAT pathway and the transcription of genes needed for production of anti-inflammatory cytokines such as IL-4. IL-4, interleukin 4; iNOS, inducible nitric oxide synthase; TNF-α, tumor necrosis factor-α; JAK, Janus kinase; STAT, signal transducer and activator of transcription; NFAT, nuclear factor of activated T-cells; NF-κB, nuclear factor kappa B; α7 nAChR, α7 nicotinic acetylcholine receptors; Gal-3, galectin-3; IGFR, insulin-like growth factor 1 (IGF-1) receptor; PPAR-γ, peroxisome proliferator- activated receptor-gamma.

The microglial α7 nAChRs have dual ionotropic/metabotropic properties and their intracellular signaling pathways that modulate inflammation do not only depend on transient ion influx (145, 155, 156). Indeed, neuronal α7 nAChRs mainly have an ionotropic function (145). The downstream metabotropic signaling pathways of microglial α7 nAChRs are different from neuronal α7 nAChRs (157). Activation of microglial α7 nAChRs induces phospholipase C and enhanced calcium release from intercellular stores which are sensitive to inositol trisphosphate (153). This process results in the inhibition of NF-κB transcriptional activity (Figure 2) (158). As a result of this inhibition the levels of pro-inflammatory cytokines are decreased (154).

It has been shown that chronic restraint stress (CRS) alters central cholinergic signaling in brain regions that have been implicated in MDD (159). Namely, CRS induces hippocampal choline acetyltransferase protein expression and decreases nuclear STAT3 signaling. CRS also augments signaling activity, IL-1β and TNF-α expression and microglial activation. Intriguingly, cholinergic stimulation with a selective α7 nAChR agonist significantly diminishes CRS-induced depressive-like behavior, neuroinflammation and neuronal damage. Moreover, activation of α7 nAChRs restores central cholinergic signaling function, inhibits TLR4-mediated inflammatory signaling and microglial activity and increases the number of regulatory T-cells in the hippocampus following stress (159).

α7 nAChR activation induces the transcriptional activity of Nrf2 (143). Previous studies especially by the Lopez group indicate that α7 nAChR mediated activation of Nrf2 elicits anti-inflammatory mechanisms in microglia (160–165). This anti-inflammatory axis might play an instrumental role in antidepressant aspects of α7 nAChR modulators. In this context, the efficacy of selective or promiscuous ligands that can activate α7 nAChR-Nrf2 pathway have been shown in depressive disorders (144, 164). One interesting example for the promiscuous ligand is Tropisetron. This ligand is a 5-HT_3_ receptor antagonist and α7 nAChR partial agonist. This serotonergic ligand has shown a great efficacy in a wide range of psychiatric disorders including MDD and schizophrenia in both experimental models and clinical trials (144, 166). The other example is RG3487 (C_15_H_19_ClN_4_O), the novel 5-HT_3_ antagonist with α7 nAChR partial agonist properties. This ligand significantly improves attentional performance in experimental models and has shown promising results in clinical trials for cognitive impairment associated with schizophrenia (142, 167).

### IL-4 Receptor Mediated Pathway

IL-4 is a multifunctional cytokine secreted by Th2 cells, mast cells, eosinophils and basophils (168, 169). IL-4 is a crucial molecule for microglia and macrophage polarization and it plays pivotal roles in brain function following neuroinflammatory insult (169). The effects of IL-4 are mediated through the IL-4 receptor α-chain. Following binding to its ligand, IL-4 receptor α-chain dimerizes either with the common γ-chain to produce the type-1 signaling complex located mainly on hematopoietic cells, or with the IL-13 receptor α 1 to produce the type-2 complex, which is expressed also on non-hematopoietic cells. The type-1 signaling complex (Figure 2) is pivotal for alternatively activated macrophages (168). Upon activation, the type-1 complex signals through JAK1 and JAK3, which phosphorylate and create docking sites for the transcription factor STAT6. This transcriptional factor then dimerizes and translocates to the cell nucleus to regulate the expression of several genes (168).

IL-4 might be protective against depression due to its ability to harness inflammation and to inhibit serotonin transporter activity. Wachholz et al. demonstrated that a decreased IL-4 responsiveness of microglia is specifically related to the development of depressive-like behavior. IL-4 deficient mice show notable augmentation of depressive-like behavior in the forced swim and tail suspension test (170). In experimental models of stress, the decline in IL-4 levels in the locus coeruleus may be involved in anxiety-like behavior and an inverse relationship between IL-4 secretion and hypothalamic-pituitary-adrenal (HPA)/sympathetic-adrenal-medullary-axes activation has been reported (171). These findings suggest that modulation of the IL-4 receptor signaling pathway is required to adapt to homeostatic mechanisms in response to stressful events (171). In addition, it has been shown that microglial IL-4 receptor pathway modulates cognitive function following neuroinflammation (172).

It is well established that adult neurogenesis in the dentate gyrus of the hippocampus is regulated by specific microglia population and potentially implicated in MDD (10, 173). Very recently, Zhang et al. showed in rodents that IL-4 driven microglia modulate stress resilience through BDNF-dependent neurogenesis (173). Their findings indicated that IL-4 driven microglia are characterized by a high expression of Arg1 which is critical in maintaining hippocampal neurogenesis and stress resistance. Decreasing Arg1^+^ microglia in the hippocampus by knocking down the microglial IL-4 receptor inhibited hippocampal neurogenesis and enhanced stress vulnerability. Indeed, Increasing Arg1^+^ microglia in the hippocampus by enhancing IL4 signaling restored hippocampal neurogenesis and the resilience to stress-induced depression (173).

Following an inflammatory insult, endogenous IL-4 can interact with its tyrosine kinase IL-4 receptor on the microglia cell surface (Figure 2). This interaction induces the production of Galectin-3 (Gal-3), prostaglandin (PG) J2 and activates STAT6. Production or activation of these molecules ultimately leads to activation of canonical transcriptional factors for microglia polarization such as PPAR-γ. The transcriptional activity of PPAR-γ can induce microglia alternative activation by decreasing the production of ROS, pro-inflammatory cytokines and suppressing the activity of NF-κB (15, 27, 174). PPAR-γ is an important and canonical transcriptional factor in the induction of anti-inflammatory signaling pathways (discussed in detail in the next section).

### Peroxisome Proliferator-Activated Receptor Gamma Mediated Pathway

Peroxisome proliferator-activated receptors (PPARs) are ligand-activated transcription factors of the nuclear hormone receptor superfamily. PPARs exist as three isoforms (α, γ, and δ/β). PPARs have a ligand binding domain and a DNA binding domain. When their endogenous or exogenous ligands bind to PPARs, they create a heterodimeric complex which recruits other co-activators including PPAR coactivator-1, PPAR-interacting protein, PPAR-binding protein, steroid receptor co-activator-1 and CREB binding protein. This complex binds to the promoter regions of specific genes that contain a regulatory element known as the peroxisome proliferator response element which either activates or transrepresses the target genes (175). In the mammalian body, PPARs control glucose metabolism, cell proliferation and differentiation (176). The fact that PPAR-γ is involved in the modulation of macrophage differentiation and activation in peripheral tissues led to studying of the role of PPAR-γ in CNS resident microglia. Several investigations indicate that PPAR-γ endogenous ligand and synthetic agonists might influence brain inflammation by inhibiting different functions related to microglial activation, such as production of inflammatory cytokines, chemokines, nitric oxide and prostaglandins (PGs) (177).

PPARγ ligands elicit anti-inflammatory and neuroprotective actions in various experimental models of neurodegenerative diseases (178). Indeed, PPARγ activation inhibits the activity of transcription factors including NF-κB, AP1, and STAT. Some studies indicate that IL-4 receptor signaling increases the endogenous level of PPAR-γ ligands such as PGJ2 and subsequently amplifies transcriptional activity of PPAR-γ which might polarize microglia phenotype toward the anti-inflammatory one (179).

The PPARγ-mediated pathway has been the subject of several pre-clinical and human studies of MDD. A low PPARγ level in the hippocampus and PFC has been associated with depressive-like behavior in mice (180). Selective agonists of PPAR-γ already have FDA approval for the treatment of type 2 diabetes and their potential antidepressant effects, through modulation of metabolism and inflammation, have been investigated in different models (181). Interestingly, the efficacy of PPAR-γ ligands has been shown in metabolic disorder to be associated with depressive-like behavior in rodents. Namely, obesity in rats results in downregulation of PPARγ in the PFC (182), meanwhile chronic treatment with pioglitazone reversed depressive-like behaviors associated with obesity in CUMS mouse model (183). There is an increased risk for obese patients with chronic low-grade inflammation to develop depression (184, 185). Also, obesity induces microglial activation and neuroinflammation that play crucial roles in the pathogenesis of depression (186).

In an interesting report Qin et al. demonstrated that CUMS can induce severe depressive-like behaviors, neuroinflammation and reduced expression of PPARγ in leptin-deficient (ob/ob) mice as compared to wild type mice. Administration of a selective PPARγ agonist, pioglitazone rectified the behavioral abnormalities and alleviated microglial pro-inflammatory cytokine levels and NF-κB activation in PFC and hippocampus (187). Other studies also investigated anti-inflammatory and antidepressant effects of PPAR-γ agonists. Li et al. aimed to explore the effects of pioglitazone on depressive-like behaviors of mice treated with LPS and elucidated the underlying mechanisms. Their findings indicated that PPAR-γ activation induces PI3K/AKT/JNK/p38 signaling pathway and counteract LPS mediated apoptosis in mice PFC (188).

Studies have provided evidence that the antidepressant-like effect of pioglitazone in the forced swim test is mediated partly through *N*-methyl-D-aspartate (NMDA) receptor signaling and nitric oxide pathway (189, 190). Cognitive impairment is a feature of both AD and psychiatric disorders. The PPARγ agonist, rosiglitazone improves hippocampus-dependent cognitive deficits. Its cognitive enhancement partly occurs through the induction of ERK cascade, a critical mediator of memory consolidation. Jahrling et al. showed that PPARγ agonism facilitated recruitment of PPARγ to pERK during memory consolidation (191). Other investigations have pointed to the involvement of NMDA receptor and nitric oxide pathway in the memory improving effects of PPARγ ligands (192, 193). These findings highlight the fact that PPARγ ligands might have therapeutic implication in MDD specifically in the patients that have memory impairments. Namely, Sepanjnia et al. showed that pioglitazone, a selective PPAR-γ agonist, is an effective and safe short-term add-on therapy to Citalopram in non-diabetic patients with MDD and was associated with a high rate of early improvement and remission (194). Taken together, these findings showcase the potential for developing new interventions that target the brain’s innate immune responses in different psychiatric disorders. However, much is still unknown about role of microglia in psychiatric disorders and why neuroinflammation is not a common phenomenon in all MDD patients (21).

### Galectin-3 Mediated Pathway

Galectins are a family of soluble β-galactoside-binding proteins found in all multicellular organisms. They act as both DAMPs in innate immunity and/or as pattern-recognition receptors that bind to pathogen-associated molecular patterns. Gal-3 has recently been implicated in studies of neuroinflammatory diseases (195). This lectin is involved in cell-cell adhesion, modulation of the brain’s innate immune response and microglial activation patterns in both physiological and pathophysiological settings. Gal-3 also mediates cell proliferation and migration (196, 197). Several studies using different approaches and methods have demonstrated both protective and deleterious effects of Gal-3 in neuroinflammatory diseases making Gal-3 an attractive target in drug discovery. Among different galectin family members, Gal-3 is unique in that in addition to the carbohydrate recognition domain, it possesses a proline and glycine-rich N-terminal domain through which it forms oligomers (195, 198). Gal-3 is expressed in epithelial cells, endothelial cells, neurons and immune cells where it is synthesized as a cytosolic protein. It can be released or secreted into the extracellular space where several bind to cell surface glycoproteins (199). Originally identified as a marker of activated macrophages, there is increasing evidence suggesting its role as a modulator of microglial phenotypes in neuroinflammation (200).

Gal-3 plays important extracellular physiological roles. It uses IL-4 dependent mechanisms to mediate microglial arborization (195). *In vitro* studies highlight the importance of the carbohydrate-binding site of extracellular Gal-3 in microglia motility and ramification. Microglia pruning of axons and synaptic terminals might involve Gal-3 (18). Intracellular Gal-3 also holds distinct roles in physiological and pathological conditions. Following inflammatory insult, endogenous IL-4 interacts with microglial IL-4 receptors, thereby increasing the production of Gal-3 and subsequently inducing the canonical transcriptional factor PPAR-γ leading to anti-inflammatory signaling (Figure 2), as described in Section “Peroxisome Proliferator-Activated Receptor Gamma Mediated Pathway” (15, 201). In neuroinflammatory events Gal-3 elicits time-dependent protective actions. For instance, a Gal-3 feedback loop is critical for IL-4-mediated alternative polarization of peripheral macrophages (200, 202). It was also shown that following neuroinflammatory insult induction of Gal-3 in proliferating resident microglia is neuroprotective (27). Additionally, Gal-3 positive proliferating microglia are the major contributing cells of neurotrophic molecules such as insulin-like growth factor 1 (IGF-1) (200) which can also enhance the effects of trophic factors such as IGF-1 through inhibition of IGF-1 endocytosis (Figure 2) (195, 200). In 2019, Rahimian et al. studied time- and context-dependent effects of Gal-3 as a neuroprotective mediator following neuroinflammation (27). We showed that Gal-3 induces an anti-inflammatory microglial phenotype through IL-4 receptor pathway. It is likely that the polarization following neuroinflammatory insults is influenced by Gal-3 binding to glycans attached to IL-4 receptors (27).

In addition to its protective actions, Gal-3 also plays a pro-inflammatory role as reported in different animal and human studies especially in neurodegenerative disorders (195). Literature suggests that microglia-derived Gal-3 is detrimental in certain neuroinflammatory conditions. Indeed, comprehensive single-cell RNA analyses of CNS immune cells in neurodegenerative conditions including AD have led to the discovery of disease-associated microglia (DAM). This subpopulation of microglia displays a distinct transcriptional and functional signature (17). Boza-Serrano et al. showed that expression of Gal-3 in DAM in a mouse model of familial AD (5xFAD). They demonstrated that in 5xFAD mice Gal-3 is expressed solely in microglia associated with amyloid-β plaques and its deletion both decreases amyloid-β burden and improves memory function. Moreover, Gal-3 was found to be a TREM2 endogenous ligand binding through its carbohydrate-binding domain (203). Gal-3 direct interaction with TLR4 receptor may be an additional mechanism by which it regulates the severity of inflammation. Burguillos et al. showed that interaction of Gal-3 with TLR4 receptors in acute phase of neuroinflammation exacerbates neural cell death and prolongs inflammation, while its ablation elicits anti-inflammatory and neuroprotective effects (204).

Emerging experimental and clinical evidence indicates that Gal-3 may also play a role in MDD (205). Recently, Stajic et al. investigated the role of Gal-3 in modulation of anxiety levels in mice (206). The finding of this study revealed contradictory effects of Gal-3 on anxiety levels in the physiological condition and following acute inflammatory challenge with LPS. Gal-3 deficiency showed clear anxiogenic effect in basal conditions that is accompanied with lower expression of brain-derived neurotrophic factor (BDNF) and GABA_A_ receptors. Gal-3 deficiency was also associated with anxiolytic response following acute administration of LPS (206). Intriguingly, the relationship between the novel inflammatory aspect of Gal-3 and depression symptom severity has been studied. In a large sample size, King et al. demonstrated higher Gal-3 levels were associated with higher levels of depressive symptoms. Their findings suggest that Gal-3 may be a new and useful inflammatory biomarker associated with depression (207). Another interesting clinical investigation showed that depression in type 1 diabetes is associated with high levels of circulating Gal-3 (208).

## Metabolic Pathways in Microglia

### Cannabinoid Signaling in Microglia

The endogenous cannabinoid (endocannabinoid) system has been implicated in synaptic communication and influences anxiety and cognition, metabolism, growth and development and response to internal and external immune insults via an array of actions mediated by their receptors (209, 210). In the CNS, endocannabinoids such as anandamide (AEA) and 2-arachidonoylglycerol (2-AG) regulate several physiological functions via two main G-protein-coupled cannabinoid receptors 1 and 2 (CB1 and CB2) (211). Endocannabinoids can also interact with several extracellular and intracellular targets such as G-protein-coupled receptor 55 (GPR55), PPARs and transient receptor potential vanilloid 1 (212). CB1 receptors are expressed in the cortex, hippocampus, cerebellum, basal ganglia and brainstem, usually at presynaptic terminals or on axons (210). They have also been reportedly found on glial cells (213, 214). CB2 receptors are much less expressed in the CNS compared to CB1 receptors, however, they have been found in the brainstem, cerebellum and hippocampus among other areas (212) and are primarily found on immune cells, astrocytes and less commonly in neurons (215). Endocannabinoids are generally synthesized post-synaptically after Ca^2+^ influx or activation of Gq/11-linked G-protein-coupled receptors; they act in a retrograde fashion influencing presynaptic cell firing (210, 215).

AEA is synthesized by phospholipase D catalyzed hydrolysis of *N*-acylphosphatidylethanolamine (216), meanwhile, 2-AG synthesis from membrane phospholipids is catalyzed by phospholipase C and diacylglycerol lipase (217). AEA degradation occurs predominantly by the enzyme fatty acid amide hydrolase (FAAH) or by cyclooxygenase (COX)-2 oxidation creating PGs (210) while 2-AG degradation occurs mostly through monoacylglycerol lipase (MAGL) but it can sometimes be oxidized by COX-2 or hydrolyzed by FAAH (210, 218). Although some processes occur due to crosstalk between cell types (219), microglia contain the complete machinery required for a functional endocannabinoid system. Rodent microglia are known to express both CB1 and CB2 (220, 221). The presence of CB1 in human microglia is controversial (211, 222), however, a few studies describe CB1 microglial expression in active multiple sclerosis plaques of postmortem human brain samples (223, 224). Microglia also produce the enzymes responsible for hydrolysis and inactivation of AEA and 2-AG.

**FIGURE 3:**
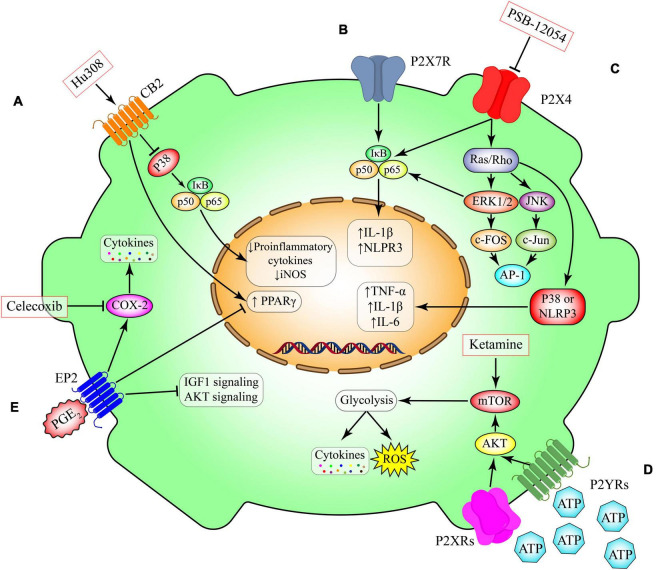
Metabolic pathways in microglia. **(A)** Activation of CB2 receptors that are expressed in non-neural cells including microglia promotes anti-inflammatory cascades through inhibition of NFκB or induction of anti-inflammatory transcriptional factor such as peroxisome proliferator-activated receptor gamma (PPAR-γ). This pathway can be induced by Hu308. **(B)** P2X7 receptor activation, induces the canonical pro-inflammatory transcriptional factors such as NFκB and subsequent production of inflammatory mediators such as IL-1 beta and NLPR3. **(C)** Activation of ligand gated ion channel P2X4 triggers the switching on two canonical pathways including NFκB and Ras/ERK/JNK. These proteins induce the production of several cytokines such as TNF-α, IL-beta and IL-6. This pathway can be inhibited by PSB-12054. **(D)** Induction of the G-protein coupled receptors P2YRs have essential roles in modulating the expression of metabolic pathways such as mTOR and their downstream glucose metabolism. The induction of glycolysis through mTOR has been implicated in production of several cytokines and chemokines. Ketamine triggers the mTOR pathways leading to induction of glycolysis. **(E)** Prostaglandin E2 (PGE 2) is a lipid mediator derived from the fatty acid arachidonic acid. Its interaction with the microglial G-protein coupled receptor EP2 induces the activity of cyclooxygenase-2 (COX-2) and inhibits several intracellular pathways including PPAR-γ, AKT and IGF1. Celecoxib is s selective COX-2 inhibitor. CB2, cannabinoid type 2 (CB2) receptor; P2X4, P2X purinoceptor 4; P2X7, P2X purinoceptor 7; P2YR, purinergic receptor P2Y; EP2, prostaglandin E2 receptor 2; PGE2, prostaglandin E2; IGF1, insulin-like growth factor 1; AKT, RAC(Rho family)-alpha serine/threonine-protein kinase; COX-2, cyclooxygenase-2; TNF-α, tumor necrosis factor-α; iNOS, inducible nitric oxide synthase; IL-1β, interleukin 1 beta; IL-6, interleukin 6; PPAR-γ, peroxisome proliferator- activated receptor-gamma; mTOR, mammalian target of rapamycin; AP-1, activator protein 1; JNK, c-Jun N-terminal kinases; NLRP3, NLR family pyrin domain containing 3; ERK, extracellular-signal-regulated-kinase; IκB, nuclear factor of kappa light polypeptide gene enhancer in B-cells inhibitor; ROS, reactive oxygen species.

The endocannabinoid system has recently been implicated as a regulator of microglial migration and activity which points to cannabinoids being a useful target for modifying microglia in pathological conditions. Reusch et al. showed that the CB2 receptor is necessary for TLR-mediated microglia activation through p38 MAPK signaling (225). Other studies have revealed that the CB2 receptor is instrumental to induce the anti-inflammatory phenotype in microglia (226, 227). Tao et al. (227) found that JWH133, a selective CB2 receptor agonist promotes the anti-inflammatory phenotype in microglia through CB2 receptor stimulated cAMP/PKA pathway (227). We and others have showed that CB2 activation can trigger the activity of canonical anti-inflammatory transcriptional factors such as PPAR-γ (Figure 3) (228–230). As discussed in previous sections, the transcriptional activity of PPAR-γ is pivotal for microglia alternative activation by diminishing the production of pro-inflammatory cytokines and inhibiting the activity of NF-κB. Following neuroinflammation, CB2 receptors are upregulated (212) which has been shown to trigger microglia migration to the site of injury/lesion (231, 232). Experimental studies elucidate that neuroinflammation produces adenosine triphosphate (ATP) (discussed in the Purinergic Signaling section), which causes 2–AG production, commencing microglia migration through activation of the CB2 receptors at the microglial leading edge (233).

The human endocannabinoid system has been implicated in MDD (234, 235). A 2019 meta-analysis revealed a very strong association of CB2rs2501432 polymorphism with depressive disorder, but not CB1rs1049353 polymorphism (236). Moreover, peripheral serum levels of AEA and 2-AG are significantly reduced in women diagnosed with MDD (237). Few studies have investigated the endocannabinoid system in postmortem human brain of psychiatric cases. Hungund et al. (238) found that CB1 receptor protein is increased in the dorsolateral PFC of depressed suicides. Moreover, using [35S]GTPgammaS binding assays which assesses coupling of G-proteins to G-protein-coupled receptors in postmortem brain, the authors found that CB1 cannabinoid signaling was increased in the same region when compared to healthy controls (238) implicating cannabinoid signaling in both depression and suicide.

The role of the endocannabinoid system has also been studied in different stress paradigms (Table 3) (239, 240). RSD of mice has been used to model depression and anxiety. This paradigm not only has stress related behavioral outcomes and impaired fear extinction but also an increase in inflammation both peripherally and in the brain. Lisboa et al. showed that stimulating CB1/2 by injecting WIN55,212-2, a non-selective agonist, daily before the RSD paradigm, reduced IL-1β mRNA in the brain but specifically in CD68^+^ activated microglia. Moreover, activation of the CB1/2 receptors before RSD repaired fear extinction and stress-related behavioral deficits (240). Although interesting, this study begs the question of whether these protective effects were mediated through the CB1 or CB2 receptor. In 2011, Zoppi et al. showed that daily pre-stress administration of arachidonyl-2′-chloroethylamide (ACEA), a selective CB1 receptor agonist, prevented upregulation of pro-inflammatory markers in the PFC of wild type mice; but not in CB1^–/–^ knockouts (239). In another study by García-Gutiérrez et al. overexpression of CB2 receptor in mice had a protective effect, providing resilience to chronic mild stress and decreased depressive-like behaviors measured by the forced swim test and novelty-suppressed feeding test. Interestingly, chronic (4-week) administration of the CB2 receptor antagonist AM630 had anti-depressant like effects in wild type mice but not those that overexpressed CB2 receptor (241). More recently, a CDS stress paradigm was used in both CB1^–/–^ knockouts and wild type littermates. Beins et al. found that CB1 knockouts mice were much more susceptible to CSD stress and mild CSD showing significant stress behaviors. Moreover, these stressed CB1^–/–^ mice had dysregulated HPA axes with insufficient glucocorticoid signaling and hyper-activated microglia (242). Interestingly, at baseline, CB1^–/–^ mice have increased expression of *Fkbp5*, a negative regulator of glucocorticoid signaling and a gene already implicated in depression (243). Overall, it seems that the endocannabinoid system serves a protective role in counteracting neuroinflammation by induction of anti-inflammatory profiles in microglia.

**TABLE 3:** Summary of cannabinoid receptors involvement in stress response.

Study	Stress paradigm	Animal model	Treatment	Major findings
(239)	Immobilization/acoustic stress (2 h/day for 4 days)	Male Swiss ICR mice (WT and CB1^–/–^ KO)	CB1 agonist: arachidonyl-2′-chloroethylamide (ACEA) (2.5 mg/kg, daily before stress, intraperitoneally)	WT: stress (1) ↑ CB1 mRNA and protein, (2) ↑ TNF-a mRNA (3) ↑ MCP-1, (4) ↑ NOS-2. ACEA pretreatment is protective against neuroinflammatory response to stress. KO: stress (1) dysregulates HPA axis, (2) ↑ TNF-a, (3) ↑ MCP-1. In sum: pretreatment with CB1 agonist is protective and CB1^–/–^ KO aggravates neuroinflammation after stress.
(240)	Repeated social defeat (2 h/night for 6 nights)	Male C57BL/6 and aggressor CD-1 mice	CB1/2 agonist WIN55,212-2 (WIN) (1 mg/kg, daily 30 min before stress, intraperitoneally)	WIN (1) ↓ stress-induced anxiety, (2) ↓ IL-1 beta in CD68^+^ microglia (3) prevented stress-induced prolonged fear response and repaired fear extinction
(241)	Chronic unpredictable mild stress (several times a day for 7–8 weeks)	Male Swiss ICR mice (WT and CB2 overexpressors)	CB2 antagonist (6-iodo-2-methyl-1-(2-morpholinoethyl)-1H-indol-3-yl) (4-methoxyphenyl)methanone (AM630). (1 mg/kg, twice daily, post-stress, for 4 weeks, intraperitoneally)	WT: stress (1) induced depression, (2) ↓ BDNF in hippocampus. CB2 overexpressor: (1) ↓ susceptibility to depression (2) no change in BDNF. Chronic AM630 treatment acted as an anti-depressant in stressed mice, protected against BDNF reduction.
(242)	Mild CSD stress (1–2 min/day for 10 days)	Male WT and B6.cg Cnr1^tm1Zim^ *Cnr1*^–/–^ KO mice Male CD-1 aggressor mice	–	KO: (1) ↑ susceptibility to mild CSD stress, (2) ↑ CD11b on microglia (3) ↑ percentage of CD11b^+^ microglia (4) ↓ microglial complexity.

*WT, wild-type; KO, knockout; CB1, cannabinoid receptor 1; CB2, cannabinoid receptor 2; ↑, increased; ↓, decreased/reduced; HPA, hypothalamic-pituitary-adrenal; MCP-1, monocyte chemoattractant protein-1; NOS-2, nitric oxide synthase-2; BDNF, brain derived neurotrophic factor.*

Rescuing cellular function as a treatment for MDD has been mainly considered for neurons but not for glial cells. However, many investigations have demonstrated the functional impairment in glia cells as mentioned throughout this review. Microglial malfunctions have been studied in many different neuroinflammatory settings (244). Research has shown that different aspects of microglia such as phagocytic activity, secretory profile and metabolic pathways can be affected by neuroinflammation. Due to this complexity, selective modulators rather than general anti-inflammatory agents might be needed to rescue microglial functions following different types of inflammatory insults (10). One interesting and promising target could be endocannabinoid system. Fine tuning of this system in microglia can open a new avenue of research in pharmacotherapy of depression although several experimental and clinical studies should be performed long before being able to design cell-specific interventions for treating MDD.

### Prostaglandin Signaling in Microglia

The PGs are a class of eicosanoids that are formed by the liberation of arachidonic acid from phospholipids and a 2-step conversion by COX, the rate-limiting enzymes (245). Two main isoforms of COX exist, COX-1 and COX-2. COX-1 is traditionally considered as a constitutive enzyme while COX-2 is inducible. However, such classification is not very precise especially in the brain where constitutive expression of COX-2 has been reported (245). One of the canonical PGs in the CNS is PGE2. It interacts with different G-protein-coupled receptors including EP1, EP2, EP3, and EP4 (Figure 3) (246). Intriguingly, elevated PGE2 in the saliva, serum and CSF of depressed patients has previously been reported (247–250). Clinical investigations also revealed that adjunctive therapy with non-steroidal anti-inflammatory drugs, known as COX inhibitors, might have therapeutic effects in a subset of MDD patients (251, 252).

Human studies have shown decreased dopamine metabolites in the CSF of MDD patients. Furthermore, acute treatment with antidepressants induces dopamine release in the medial PFC (253, 254). These findings imply that activation of the mesocortical dopaminergic pathway has anti-depressive properties. In this context, the role of PGE2 has been studied in several experimental paradigms. It has been shown that EP1-deficient mice showed hyperdopaminergic activity, leading to impulsive behaviors under acute social and environmental stress (255). It is noteworthy that EP1 is located on GABAergic terminals on midbrain dopamine neurons and electrophysiological recording indicates that EP1 stimulation potentiates inhibitory synaptic inputs to these neurons (256). These findings suggest that PGE2-EP1 signaling suppresses midbrain dopamine neurons and regulates impulsive behaviors under acute stress (257). However, the mechanisms underlying involvement of the mesocortical dopaminergic pathway in vulnerability to repeated stress is unknown. Indeed, PGE2-EP1 signaling attenuates mesocortical dopaminergic pathway, leading to susceptibility of mice to RSD (254). Analyses of c-Fos expression of ventral tegmental area dopamine neurons and dopamine turnover in medial PFC showed that the mesocortical dopaminergic pathway is activated upon social defeat and attenuated with repetition of social defeat in wild-type mice. EP1 deficiency abolished such repeated stress-induced attenuation of mesocortical dopaminergic pathway (254).

Intriguingly, PGE2 acting on striatal medium spiny neurons has been suggested to elicit a negative affective state in response to inflammatory or social stress (254, 258). Our knowledge about the source of PGE2 was limited until recently. Klawonn et al. revealed that microglial PG signaling as critical for inflammation-induced aversion and is a potential mechanism by which different types of stressors may converge to produce a negative affective state and potentially depression (259). Indeed, these findings demonstrated that striatal microglial activation induces negative affect and both IL-6 and PG dependent signaling in microglia is critical for inflammation induced aversion. Chemogenetic activation of striatal microglia induces an aversive affective state while chemogenetic inhibition of microglia blocks inflammation induced aversion. Microglial IL-6 signaling and PG synthesis regulate affective state and finally PGE2 from activated microglia reduces the excitability of striatal neurons (259). In agreement with these findings, different investigations indicate that low-dose aspirin, which primarily inhibits COX-1 and consequently PG production, reduces the risk of depression (260). The inducible form of COX, COX2, might also be involved in the MDD pathology, since the COX-2 selective inhibitor celecoxib has beneficial effects in subset of depressed patients (83, 261). Since strong microglial COX-1 expression is complemented by COX-2 in response to chronic inflammation and stress (262, 263), both enzymes could contribute to depressive symptoms at different stages of the disease or in distinct patient groups (259).

### Mammalian Target of Rapamycin Signaling in Microglia

The mammalian target of rapamycin (mTOR), the evolutionarily conserved serine/threonine protein kinase, may be activated by phosphorylation in response to growth factors (such as BDNF), mitogens and stress (264, 265). The mTOR signaling pathway plays a fundamental role in the regulation of protein synthesis, energy metabolism, lipid metabolism, cell growth and autophagy (266). In the CNS, mTOR is also involved in axonal sprouting, axonal regeneration and myelination, ionic and receptor channel expression, dendritic spine growth, as well as astrocyte migration and proliferation. mTOR-regulated processes in the brain influence neuronal excitability, neuronal survival, synaptic and behavioral plasticity, cognition, feeding, and control of circadian rhythm (265). In recent years, special attention has been given to the role of mTOR signaling in MDD. Several investigations have reported decreased brain mTOR activation in animal models of depression (267). One of the most studied models is CUS, which mimics several behavioral and neurochemical alterations that occur in depressed individuals (268). Rodents exposed to CUS exhibit depressive-like behaviors associated with a reduction in phosphorylation levels of mTOR and its downstream signaling components, such as phosphor-p70S6K, in the PFC, hippocampus and amygdala (268, 269). Regarding the anti-depressive role of the mTOR pathway, an elegant study by Li et al. revealed a single dose of ketamine can activate mTOR, resulting in increased PFC synaptic protein expression within 2 h and increased dendritic spine density and synaptic activity within 24 h (267). Importantly, clinical evidence also confirms the role of mTOR signaling in MDD pathology (270). It has been shown that mTOR, p70S6K, eIF4B, and p-eIF4B protein expression in PFC of deceased MDD subjects were reduced when compared with controls, indicating a deficit in mTOR-dependent signaling leading to impairment in its downstream targets that control translation of synaptic proteins (270).

Intriguingly, mTOR signaling can regulate several aspects of microglial function such as phagocytosis and cell survival (271). For instance, inhibition of mTOR diminishes the viability of primary cultured microglia (272), whereas induction of mTOR activity by inhibiting its upstream suppressor, tuberous sclerosis 1, enhanced phagocytosis in microglia (271, 273). Furthermore, microglial-specific inhibition of mTOR pathway decreases proinflammatory cytokines and chemokines (274). More recently, it has been shown that mTOR-mediated metabolic reprogramming shapes distinct microglial functions in response to LPS and ATP (271). Hu et al. showed that both LPS and ATP induced rapid activation of mTOR and glycolysis in microglia. Blocking either glucose metabolism or mTOR activity inhibits glycolysis significantly and mitigates LPS-induced production of proinflammatory cytokines, indicating that mTOR-driven glycolysis is required for the proinflammatory responses of LPS-primed microglia (271). Additionally, blocking mTOR activity not only inhibits glycolysis but also suppresses BDNF and TNF-α production in ATP-activated microglia, suggesting the critical role of mTOR in tuning microglia function (271). Better understanding of the metabolic regulation of microglia help us to manipulate and control the activity of microglia following different neuroinflammatory insults. The distinct metabolic adaptation in microglia in response to different stressors may provide diverse approaches to target microglia at different states and restrain microglia-triggered neuroinflammation in neuropsychiatric disorders (271).

Our group provided the first evidence of increased microglial activation in dorsal ACC white matter of depressed suicides. Although total density of Iba1^+^ microglia remained unchanged between depressed suicides and matched controls, the ratio of primed to ramified microglia was significantly increased in depressed suicides (10, 38). The mechanisms underlying microglial priming are unknown. However, it has been shown that the mTOR signaling pathway plays a crucial role in microglial priming during aging. Keane et al. were the first to show that microglia from aged mice have upregulated mTOR complex 1 signaling controlling translation and protein levels of inflammatory mediators (275). Genetic ablation of mTOR signaling in mouse microglia caused an NF-κB–dependent upregulation of priming genes at the mRNA level. However, mice displayed reduced cytokine protein levels, lessened microglial activation and milder sickness behavior. Similar changes were present in aged human microglia revealing that upregulation of mTOR-dependent translation is an essential aspect of microglia priming in aging (275). It is possible that abnormalities in microglial mTOR signaling are involved in the emergence of the primed microglial phenotype in MDD.

### Purinergic Signaling in Microglia

ATP, adenosine di-phosphate (ADP) and adenosine are molecules that are involved in purinergic signaling through P1, P2X, and P2Y receptors. G-protein-coupled P1 receptors are selective for adenosine and there are four subtypes A_1_, A_2A_, A_2B_, and A_3_. Meanwhile P2 receptors are activated by both ATP and ADP. There are two types of P2 receptors, namely P2X ionotropic channels and P2Y G-protein-coupled receptors (Figure 3). P2X receptors have seven subtypes while P2Y have eight subtypes. Microglia express several purinergic receptors (276) including: P2X1, P2X4, P2X7 (277), P2Y4, P2Y7, P2Y6, P2Y11 P2Y12, P2Y13, A_1_, A_2A_ and A_2B_ (278). Notably, adenosine binding to A_2A_ has been shown to mediate microglial process retraction (279). Besides microglial motility, purinergic signaling is important in many other processes such as neurodevelopment and neuron-glial crosstalk and inflammation (280). Interestingly, many aspects of the purinergic signaling system have been implicated in depression (281) and here, we will focus on the receptors expressed by microglia (Table 4).

**TABLE 4:** Summary of microglial purinergic signal transduction.

Receptor	Type	Ligand	Role
A_2A_	G-protein coupled receptor	Adenosine	Process retraction (279)
P2Y1	G protein coupled receptor	ADP	Migration and chemotaxis (285, 286)
P2Y6	G-protein coupled receptor	Uridine	Mediates cytokine secretion (295)
P2Y12	G-protein coupled receptor	ADP	Specifically expressed in microglia (288). Migration and chemotaxis (285, 286, 289). Neuron-microglia interactions (287, 288, 293). Regulates innate fear behaviors (293)
P2X4	Ligand gated ion channel	ATP	Phenotype switch to pro-inflammatory (276) Drives release of BDNF (294)
P2X7	Ligand gated ion channel	ATP	Mediates cytokine secretion (295) Oligomerization of NLR3P inflammasome (296)

*BDNF, brain derived neurotrophic factor; NLR3P, NLR family pyrin domain containing 3.*

ATP is released by injured cells into the extracellular space acting as DAMPs and a chemoattractant for microglial processes (233, 282, 283). Experimentally injected ATP causes microglia chemotaxis and process extension toward the site of injection (284). This process occurs through the G-protein-coupled P2Y (P2Y1R and P2Y12R) receptors (285, 286). P2Y12R is involved in long-range communication between neurons and microglia (287, 288), as well as microglia chemotaxis preceding phagocytosis (288). Additionally, P2Y12R acts as a marker for healthy microglia and is downregulated in the active pro-inflammatory phenotype in mice (289). There seems to be a species difference in the expression of P2Y12R as human brain resection from epileptic temporal lobe shows PY212R in both ramified and amoeboid microglia (290). Furthermore, the same tissue shows ADP stimulating process retraction (291) conversely to process extension in mouse microglia (284, 289). Although similar methods of comparative investigation have not yet been applied to all purinergic signaling, these findings alone suggest that they may differ greatly between species, providing necessary context to rodent studies. Loss of P2Y12R is associated with alteration in recognition and social memory as well as anxiety-like behavior in adult mice (292). Also, microglial P2Y12R regulates neural excitability and fear behaviors in developing and adult mice (293). Intriguingly, a human postmortem study found increased levels of P2Y12R protein in MDD microglia from four different brain regions, suggesting more resting state microglia in MDD and contradicting the neuroinflammation hypothesis (98).

Activation of microglial P2X4R by ATP (Figure 3) leads to a phenotype switch to pro-inflammatory state (276). Interestingly, in an ischemic stroke model, deletion of P2X4R from myeloid cells was shown to be protective in mice by inhibition of excessive pro-inflammatory cytokine release. However, after 30 days, the mice developed depressive-like phenotypes possibly due to missing P2X4R’s crucial role in BDNF release (294).

Once microglia are activated, cytokine secretion is mediated by P2Y6R (295) and P2X7R. Activation of P2X7R by ATP acting as DAMPs signal leads to oligomerization of NLR3P inflammasomes (Figure 3) (296). NLR3P inflammasome cleavage of pro-caspase 1 into caspase 1 results in the production of IL1-β from these activated microglia in situations of neuroinflammation such as in depression (297–299). In fact, a 2017 study showed that a 3-week CUS paradigm induced a depressive phenotype, increased extracellular ATP leading to P2X7R activation; this was inhibited by chronic treatment with P2X7R antagonists Brilliant Blue G (BBG) and A438079. Furthermore, P2X7R null mice that underwent the CUS did not develop a depressive phenotype irrespective of sex (300). In fact, the antidepressant phenotype of P2X7R deletion (301, 302) might be mediated by increased neurogenesis and serotonin bioavailability in the hippocampus (303). In humans, studies have suggested that polymorphisms Gln460Arg (rs2230912) (304) and His155Tyr (rs208294) in the *P2RX7* gene are associated with depression and other mood disorders (305, 306). However, a 2014 meta-analysis has contradicted these findings (307).

In the healthy brain, P2X7R is not activated by physiological ATP levels, in fact, P2X7R activation only occurs when ATP acts as DAMPs (308). Intriguingly, P2X7R antagonists that cross the BBB are in clinical trials with the hopes of using them as adjunct or monotherapy for depression caused by neuroinflammation (309, 310). Interestingly, cholesterol and other lipids like phosphatidylglycerol and sphingomyelin modulate the function of P2X7R (311). Our lab and others have found altered brain lipids in psychopathology (312) and depression (313–315) suggesting lipid control of P2X7R should be further investigated. Generally, lipids in the cell membrane organize position and function of proteins. They can be also released from the membrane acting as messengers transmitting signals (316). Lipid composition of the brain affects cognition, perception and mood most likely by influencing neuroinflammation and neurogenesis (317). Moreover, decreased polyunsaturated fatty acids seem to be the most frequently implicated lipid change in depression (313, 317) and they are known in regulating CB1 receptor (317) and P2X7R (311, 318). Interestingly, dietary supplementation with fish oil rich in polyunsaturated fats decreases depressive-like behavior in LPS treated mice and suppresses activation of the NLRP3 inflammasome and P2X7R (318). Meanwhile, sphingolipids have been implicated in both the etiology of MDD and the beneficial effects of antidepressants (316, 319). In addition to diet (320), lipid composition in the brain can be altered by environmental factors including exercise (321) and medications (322), making them promising targets for treatment (319).

To our knowledge, there is a paucity of studies on the role of microglial adenosine receptors in depression. However, A1 receptor activation generally inhibits serotonin release in the hippocampus, while A2 receptor activation promotes the release of this neurotransmitter (323). Moreover, A2 receptors have been found to be involved in the dysregulation of the HPA axis observed in depression (324). Furthermore, a 2021 study showed that 14 days of restraint stress induced depression and anxiety in the mice with a neuroinflammatory phenotype. The authors discovered increased expression of P2X7R and A_2A_ receptor in the hippocampus and prefrontal cortex. Next, they showed that BBG a P2X7R-selective antagonist and caffeine an A_2A_ receptor antagonist attenuated the depressive phenotype (325).

### Iron Metabolism in Microglia

Iron metabolism is crucial for normal functioning of the brain, including myelination by oligodendrocytes and neurotransmitter synthesis (serotonin, dopamine, and norepinephrine) (326). Transferrin, a glycoprotein is mainly responsible for the movement of iron through the body and has high affinity to Fe^3+^ (ferric iron) (327). Movement of iron from the periphery into the brain occurs through the BBB. Transferrin receptor 1 (TfR1) on endothelial cells mediates the uptake of transferrin bound iron (Tf-Fe) into the brain, while also regulating the return of iron-depleted transferrin back to the blood (326, 328). Tf-Fe is packaged into endosomes and then released into the brain through ferroportin 1 (Fp1) as Fe^2+^ where it is oxidized to Fe^3+^ through ferroxidases hephaestin or ceruloplasmin (Cp) (326, 329). Fp1 levels are controlled by hepcidin (HepC) which binds to Fp1 leading to its internalization and subsequent degradation (330). The metabolism of iron in the brain is highly controlled as it is extremely important for proper cell functioning, however, iron overload can be extremely toxic and cause cell death through oxidative stress (329). In the brain, iron is distributed heterogeneously with concentration varying by region (331, 332) and cell type (333). Iron is mainly distributed in the hippocampus (331) and the basal ganglia stored in ferritin (334) or neuromelanin (335). Presumably all cell types can uptake iron through either Tf-Fe or non-transferrin bound pathways. Neurons and astrocytes uptake Tf-Fe through TfR1 and divalent metal transporter 1 (DMT1). Oligodendrocytes uptake iron through the TfR1/DMT1 pathway only in immature states; once they mature, iron enters through heavy (H-) chain ferritin binding to H-ferritin receptor on the cell membrane (336). Oligodendrocytes have very high iron necessity for their normal functioning and proper myelination (333, 336). Microglia have two different ways of regulating iron influx; the first being through Tf-Fe uptake via the TfR1/DMT1 pathway while the second is uptake of non-Tf-Fe through transferrin-independent mechanisms (326). In healthy brain, iron usually travels bound to transferrin, which is produced by oligodendrocytes and ChP, however, it is only secreted by the latter (337).

Pathogens such as bacteria utilize iron from the host to replicate. To protect the host, immune cells are programmed to reduce iron availability to prevent further infiltration by the invader (338). This happens through increased production of HepC in hepatocytes, stimulated by IL-6 release from immune cells (339) which reduces the export of iron into the blood by negative regulation of ferroportin (340). It has been shown that different activation status in microglia prefer one iron intake pathway over the other (341). In neuroinflammatory conditions, iron is sequestered in pro-inflammatory microglia and neurons through non-transferrin bound pathways (342). *In vitro* LPS stimulation of microglia results in downregulation of TfR1 (338, 343) and ferroportin (338) and upregulation of DMT1 and ferritin (344). Under this condition, iron accumulates intracellularly and secretion of inflammatory cytokines and metalloproteinases is increased (343). Conversely, when microglia are stimulated with IL-4, an anti-inflammatory phenotype of microglia prefers Tf-Fe uptake and increases cellular transferrin receptor levels (344).

The WHO estimates that 37% of females are iron deficient globally, while males are rarely diagnosed (345). Both iron deficiency and depression were in the top disorders for most years lived with disability globally (346). Interestingly, females are twice as more likely to be diagnosed with MDD. Recent literature has implicated iron dysregulation, specifically iron deficiency, in the pathology of depression (347–351). Indeed, a recent study found that depressed individuals are 3 times more likely than healthy individuals to have hypoferritinemia (low iron storage) and acquired hypotransferrinemia (decreased levels of protein transferrin) (351). The fact that iron is a co-factor for rate limiting steps of serotonin, dopamine and norepinephrine synthesis and neuronal uptake further implicates involvement of iron metabolism in the etiology of depression (351, 352). Whether iron deficiency detected in the periphery is adequately representative of iron deficiency in the brain is still under speculation due to the tight control of iron metabolism in the brain (352). However, a 2007 study by Vostrikov et al., reported significantly reduced numbers of oligodendrocytes in layer 3 of BA9 in the PFC of postmortem samples from MDD patients (353). Given that oligodendrocytes hold the most iron in the brain, it is reasonable to infer decreased iron levels in postmortem MDD brain. Moreover, research from our lab has implicated oligodendrocyte precursor cell dysregulation with MDD (354). Iron deficiency has been shown to lead to hypomyelination in both animals and humans (355). A 2018 study revealed that developmental iron deficiency kept not only oligodendrocyte lineage cells in immature states but also other glia cells. Interestingly, iron deficiency inhibits microglial inflammatory cytokine secretion after LPS stimulation (356). Additionally, it has been shown that microglia constitute the primary source of iron to oligodendrocyte precursor cells during myelination (357).

Considering that microglia are involved in oligodendrocyte differentiation and myelin repair (358), it is a logical next step to investigate the role of iron metabolism in microglia and the relationship to depression. Few studies have investigated the role of microglia iron metabolism and depression and the ones that examined this relationship in animals found a different trend than the aforementioned human studies. Gao et al. showed that CSD stress resulted in depressive phenotype, increased microglial activation, increased iron, decreased Fp1 and increased ferritin, DMT1 and HepC in the hippocampus (359). Similarly, Jiao et al. found that mice with CUMS-induced behavioral despair were found to have increased brain iron, increased inflammation and Fe^2+^ levels in the hippocampus. Genes involved in ferroptosis were differentially regulated; specifically, glutathione peroxidase (GPX4, necessary for repairing oxidative damage) was downregulated after CUMS suggesting a role of ferroptosis in the phenotype of depression. In addition, this study also showed that fluoxetine not only resolved depressive behaviors but also restored normal ferroptosis signaling (360). Experimental models also display an increase in iron load in the brain which results in anxiety symptoms. A study by Texel et al. shows that Cp knockout mice have iron overload in the periphery, but lower levels of iron in the hippocampus accompanied by decreased levels of 5-HT, norepinephrine and BDNF (361). In another study, Pellegrino et al. showed that knockout of transferrin receptor 2 leads to iron overload in the brain, dysregulation of microglia activation status and increased anxiety levels in the animals (362). Overall, regulation of iron metabolism by microglia is an important factor in brain physiology and pathophysiology. Future studies are needed to investigate the postmortem brain of depressed individuals to better understand the link between iron metabolism abnormalities in brain innate immune system and MDD pathology.

## Sexual Dimorphism in Microglial Response in Healthy Brain and Following Stress

In recent years, special attention has also been given to the sexually dimorphic role of microglia in both healthy neurodevelopmental and diseased neurodegenerative brains (15). Sex differences have been reported in distribution, structure, function, transcriptomic and proteomic profiles of microglia in both physiological and neuroinflammatory conditions (10). Evidence demonstrates that microglia play an instrumental role in the sexually dimorphic differentiation of the developing brain (363). Interestingly, the number and phenotype of microglia differ in many regions of the brain when comparing male and female rodents (364). Brain areas such as the preoptic area, cerebral cortex and the amygdala and hippocampus have been reported to have microglial sexual dissimilarity (363–366). Importantly, the postnatal sexual dimorphism persists into the adult brain. These marked differences may lead to development of distinct, sex-dependent microglia inflammatory responses in pathological conditions. In fact, sex differences in microglial activation patterns following neuroinflammation have been reported by different groups (367, 368). However, whether the observed differences are exclusively hormone-dependent and/or stem from distinct developmental mechanisms remains to be elucidated. The analyses of the different anti- and pro-inflammatory signaling events reveal that they differ in males and females.

Several observations suggest marked sex differences in the microglial activation patterns following stress. In a pioneer study, Bollinger et al. revealed differential effects of stress on microglial activation in female and male medial PFC (369). Importantly, dysfunction of this brain area is implicated in MDD pathology. It has been shown that chronic stress affects the medial PFC in a sex-dependent manner and impairs prefrontal mediated behaviors in males and females (369). Unstressed female rats show a greater proportion of primed to ramified microglia relative to males, alongside increased CX3CL1-CX3CR1 levels. In addition, acute stress and CRS diminished the ratio of primed to ramified microglia and microglial CD40 expression in females but not in males (369). Another intriguing study revealed that the expression of genes related to cellular stress, neuroimmune state and neuron-microglia communication varied between unstressed male and female rats in a region-specific manner (370). Namely, in the dorsal hippocampus, chronic stress increased immune markers expression in males but not females. It is noteworthy that the type of stress can also mediate sex-specific innate immune response. For instance, acute stress increased microglia-associated transcripts in basolateral amygdala in males, whereas chronic stress altered immune factor expression in basolateral amygdala more broadly in females (370). It was recently shown that chronic stress induces different neurobiological adaptations in the PFC of male and female mice. CUS causes behavioral changes and microglia-mediated neuronal remodeling only in the frontal cortex of male mice (371). Fluorescence-activated cell sorting and gene expression analyses by Woodburn et al. indicate that CUS increased expression of markers of phagocytosis only in male PFC microglia (371). Overall, these findings suggest that sex differences might impact microglia pro- and anti-inflammatory signaling pathways, leading to different outcomes.

## Conclusion

Cytokine research in MDD faces several difficulties, such as conflicting results and high variability of cytokine levels within samples. Several studies have only reported serum or plasma cytokine levels without taking into account potential confounding factors such as age, body weight, smoking, alcohol consumption and medication, which can all influence the plasma content of cytokines (372, 373). These limitations imply that serum levels of cytokines may not reflect their levels in the brain and, therefore, may not reflect pathology (373, 374). Direction of causality is another important factor that is not clear in the cytokine research in psychiatric disorders. Although there is strong evidence for the involvement of cytokines in the pathology of MDD, the direction of this relationship has not yet been clarified with certainty. For instance, the alteration of cytokine levels might also be the consequence of a psychiatric disorder. Factors such as antidepressants or body mass index (that could change as a result of the disorder) might cause fluctuation in cytokine levels (373, 375).

An association between neuroinflammation and MDD has been reported by multiple studies (10) and it is well-established that inflammatory markers are increased in a subset of MDD patients (84, 85, 376) but not all (10, 21). Although microglial abnormalities have been reported in MDD, no link has been made with specific diagnostic categories. It is noteworthy that recent attempts at targeting inflammation as a new therapy for MDD have not been very promising. In fact, it appears that anti-inflammatory treatment options may only be effective in patients who show signs of increased peripheral inflammation along with depressive behaviors (83, 84). Namely, it has been shown that antagonizing TNF-α as a therapy may only prove useful in patients with high baseline inflammatory biomarkers (85). With similar uncertainty, general immunosuppressants and anti-inflammatory agents such as minocycline and non-steroidal anti-inflammatory drugs that were promising in animal studies (10) have failed in treating neuropsychiatric disorders probably due to their non-selective dampening of the immune response. Targeting of pro- or anti-inflammatory brain cytokines for developing new pharmacotherapeutics is not easy. As discussed in detail in this review, these cytokines have essential physiological actions and blockade of their signaling might influence several crucial processes such as cell survival and neural plasticity. Furthermore, the diversity of brain cell types that produce cytokines makes it difficult to target their signaling in a neuroinflammatory context. Finally, different receptor subtypes have different roles following neuroinflammation (e.g., TNF-α and IL-6 receptors).

It is becoming increasingly obvious that microglia act as a double-edged sword in the etiopathology of MDD. On one hand, some experimental studies both *in vitro* and *in vivo* have linked neuronal damage with the inflammatory phenotype of microglia releasing neurotoxic mediators and ROS (377). On the other hand, other investigations have reported the beneficial roles of microglial anti-inflammatory phenotype in neuronal regeneration and neurogenesis. Whether microglia play a positive or negative role depends on several factors including type of neuroinflammatory insult, brain region, time, sex and age. Indeed, heterogeneity of microglia and their context-dependent properties warrant more research to gain a clearer understanding of this dynamic cell type before new therapeutics can be successfully developed for psychiatric disorders such as MDD.

## Author Contributions

RR, CB, and NM conceived the review. RR, CB, and RC wrote the manuscript. NM supervised the project and revised the manuscript. All authors read and agreed with the final version of the manuscript.

## Conflict of Interest

The authors declare that the research was conducted in the absence of any commercial or financial relationships that could be construed as a potential conflict of interest.

## Publisher’s Note

All claims expressed in this article are solely those of the authors and do not necessarily represent those of their affiliated organizations, or those of the publisher, the editors and the reviewers. Any product that may be evaluated in this article, or claim that may be made by its manufacturer, is not guaranteed or endorsed by the publisher.

## Abbreviations

MDD: major depressive disorder
TNF- α: tumor necrosis factor-alpha
IL: interleukin
RA: rheumatoid arthritis
TGF- β: transforming growth factor β
PPAR- γ: peroxisome proliferator-activated receptors-gamma
CNS: central nervous system
NF κ B: nuclear factor kappa-light-chain-enhancer of activated B cells
BBB: blood–brain barrier
CUS: chronic unpredictable stress
TNF- α: tumor necrosis factor-alpha
ACC: anterior cingulate cortex
LPS: lipopolysaccharide
CSD: chronic social defeat
ROS: reactive oxygen species
CUMS: chronic unpredictable mild stress
TLR: toll-like receptor
TNFR1: TNF- α Receptor 1
TNFR2: TNF- α Receptor 2
MAPK: mitogen-activated protein kinases
CSF: cerebrospinal fluid
HPA: hypothalamic-pituitary-adrenal
gp130: glycoprotein 130
RSD: repeated social defeat
IL-6R: IL-6 receptor
DAMPs: danger-associated molecular patterns
MyD88: myeloid differentiation primary response 88
AD: Alzheimer’s disease
PFC: prefrontal cortex
IFN- γ: interferon-gamma
JAK: janus kinase
STAT: signal transducer and activator of transcription
ERK: extracellular-signal-regulated-kinase
SSRIs: selective serotonin reuptake inhibitors
ChP: choroid plexus
α 7 nAChR: Alpha-7 nicotinic acetylcholine receptor
Nrf2: nuclear factor-erythroid factor 2-related factor 2
CRS: chronic restraint stress
Gal-3: galectin-3
PG: prostaglandin
PPARs: peroxisome proliferator-activated receptors
NMDA: *N*-methyl-D-aspartate
IGF-1: insulin-like growth factor 1
DAM: disease-associated microglia
BDNF: brain-derived neurotrophic factor
endocannabinoid: endogenous cannabinoid
AEA: anandamide
2-AG: 2-arachidonoylglycerol
CB1: cannabinoid receptor 1
CB2: cannabinoid receptor 2
GPR55: G-protein -coupled receptor 55
FAAH: fatty acid amide hydrolase
COX: cyclooxygenase
MAGL: monoacylglycerol lipase
ATP: adenosine triphosphate
ACEA: arachidonyl-2 ′ -chloroethylamide
mTOR: mammalian target of rapamycin
ADP: adenosine di-phosphate
BBG: Brilliant Blue G
Tf-Fe: transferrin bound iron
Fp1: ferroportin 1
Cp: ceruloplasmin
HepC: hepcidin.
